# A Blockchain-Based Dynamic Consent Architecture to Support Clinical Genomic Data Sharing (ConsentChain): Proof-of-Concept Study

**DOI:** 10.2196/27816

**Published:** 2021-11-03

**Authors:** Faisal Albalwy, Andrew Brass, Angela Davies

**Affiliations:** 1 Department of Computer Science University of Manchester Manchester United Kingdom; 2 Department of Computer Science College of Computer Science and Engineering Taibah University Madinah Saudi Arabia; 3 Division of Informatics, Imaging and Data Sciences University of Manchester Manchester United Kingdom

**Keywords:** blockchain, smart contracts, dynamic consent, clinical genomics, data sharing

## Abstract

**Background:**

In clinical genomics, sharing of rare genetic disease information between genetic databases and laboratories is essential to determine the pathogenic significance of variants to enable the diagnosis of rare genetic diseases. Significant concerns regarding data governance and security have reduced this sharing in practice. Blockchain could provide a secure method for sharing genomic data between involved parties and thus help overcome some of these issues.

**Objective:**

This study aims to contribute to the growing knowledge of the potential role of blockchain technology in supporting the sharing of clinical genomic data by describing blockchain-based dynamic consent architecture to support clinical genomic data sharing and provide a proof-of-concept implementation, called ConsentChain, for the architecture to explore its performance.

**Methods:**

The ConsentChain requirements were captured from a patient forum to identify security and consent concerns. The ConsentChain was developed on the Ethereum platform, in which smart contracts were used to model the actions of patients, who may provide or withdraw consent to share their data; the data creator, who collects and stores patient data; and the data requester, who needs to query and access the patient data. A detailed analysis was undertaken of the ConsentChain performance as a function of the number of transactions processed by the system.

**Results:**

We describe ConsentChain, a blockchain-based system that provides a web portal interface to support clinical genomic sharing. ConsentChain allows patients to grant or withdraw data requester access and allows data requesters to query and submit access to data stored in a secure off-chain database. We also developed an ontology model to represent patient consent elements into machine-readable codes to automate the consent and data access processes.

**Conclusions:**

Blockchains and smart contracts can provide an efficient and scalable mechanism to support dynamic consent functionality and address some of the barriers that inhibit genomic data sharing. However, they are not a complete answer, and a number of issues still need to be addressed before such systems can be deployed in practice, particularly in relation to verifying user credentials.

## Introduction

### Overview

With the advent of fast and effective next-generation sequencing technologies, unlinked and dispersed genomic data have emerged as a major challenge in diagnosing rare diseases. The molecular diagnosis of a rare disease involves comparing a patient’s genetic variant data with the variants of others with similar diseases in a large population. Therefore, sharing of data between genetic databases and laboratories is essential to identify overlapping results and for determining the pathogenic significance of variants to enable the diagnosis of rare genetic diseases.

One of the most common challenges to be overcome is that genomic data are often kept in centralized restricted-access repositories because of privacy and security concerns [1-7]; therefore, the data are difficult to locate or unavailable outside of the laboratories that own them. An in-depth qualitative study has revealed that current approaches to genomic data access and sharing through restricted-access repositories are time consuming and difficult and emphasized that the availability, discoverability, and accessibility of genomic data are bottlenecks to facilitating genomic data sharing [8]. There are also further challenges that hinder the large-scale sharing of genomic data, including a lack of time and the resources required to obtain consent to share [9], insufficient resources and infrastructure to track and recontact patients [10,11], lack of interoperability [1,2,12,13], and ethical issues [1,13-15].

Some of the above-mentioned challenges are the result of adopting centralized architectures for storing, sharing, and accessing genomic data. In such architectures, the data are stored in centralized databases and accessed through controlled access mechanisms. Although this approach to the gathering and management of genomic data has proven successful in the past, studies have revealed that such centralized architectures fail to properly address the growing demand for accessing genomic data [16,17]. This is concerning because the discoverability, availability, and accessibility of genomic data are essential for enabling the diagnosis of rare genetic diseases [8,18].

Various solutions to the challenges associated with the centralized storage of genomic data have been proposed. For example, federated data storage systems have been proposed to support genomic data sharing. The GA4GH Beacon Project [19] and i2b2 Data Sharing Network [20] are examples of such systems. Both use a federated network to connect institutions’ genomic databases, which enables them to process queries concerning the presence of genetic variants and traits. This also reduces the cost of genomic data transfers and allows institutions to maintain data control [21]. However, such systems have some drawbacks, including their failure to support complex queries, limitations to research institutions and hospitals, nonallowance of patient engagement in contributing or controlling their genomic data, and lack of decentralized governance [21,22].

Decentralized and distributed technologies have been suggested as a potential solution to promote genomic data sharing [23,24]. One emerging example of such a technology is blockchain technology. As decentralized and distributed technology, blockchain technology has many appealing properties, such as data integrity and accountability, that could be used to improve the integrity, discoverability, and accessibility of genomic data, thereby moving toward a new trusted infrastructure to support the promotion of genomic data sharing. This paper proposes blockchain-based dynamic consent architecture to support genomic data sharing. We present some design considerations and describe a proof-of-concept implementation for the proposed architecture called ConsentChain. The source code is available on Mendeley data [25] under the MIT license.

### Background

#### Blockchain

##### Overview

A blockchain is a protocol that enables a network of computers, known as nodes, to maintain a shared database called a ledger, without the need for complete trust between the network’s nodes [26]. It was originally developed as the underlying infrastructure for the peer-to-peer electronic cash system Bitcoin in 2009 [27]. Other blockchain platforms, including Ethereum [28] and Hyperledger Fabric [29], have emerged as the next generation of blockchain technology and implemented the concept of smart contracts, which was first introduced by Nick Szabo in the 1990s to build a digital relationship between 2 parties over computer networks [30]. In blockchain, a smart contract is a computer program that is stored, executed, and verified in the blockchain according to predefined conditions without the need for any trusted-third party [31]. The result of smart contract execution is a transaction recorded on a blockchain [28]. Ethereum smart contracts are written using high-level programming languages, such as Solidity and Vyper; therefore, they are vulnerable to coding bugs and malicious flaws [32].

##### Blockchain Architecture

A blockchain consists of 2 main components: a peer-to-peer network and a distributed ledger.

**Figure 1:**
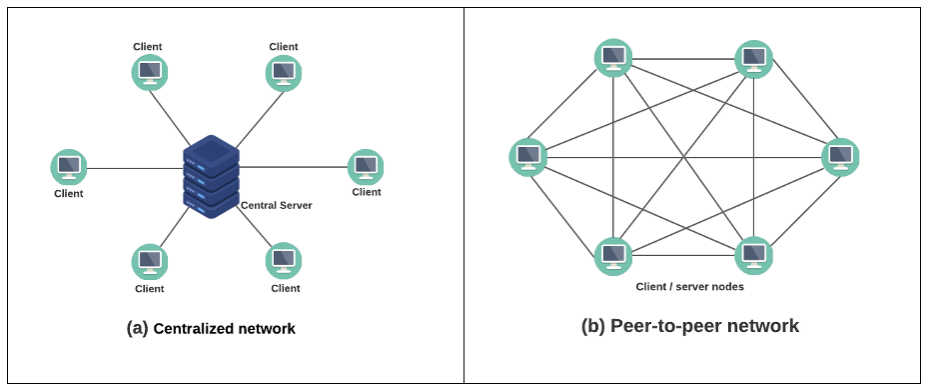
The architectures of centralized and peer-to-peer networks.

**Figure 2:**
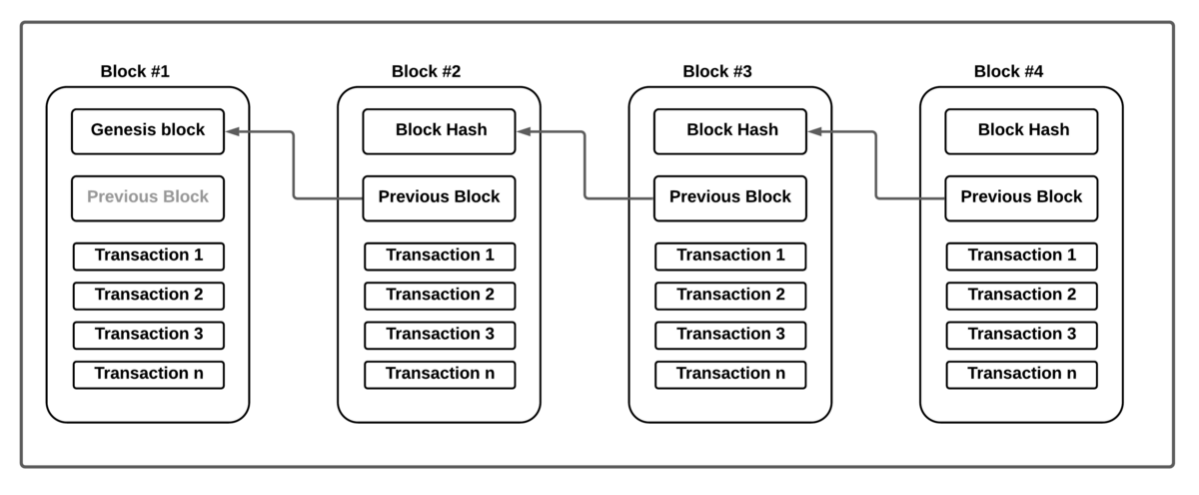
Simplified blockchain concept.

- Peer-to-peer network: understanding peer-to-peer networks is essential for understanding blockchains because, at its core, a blockchain is a peer-to-peer network. As stated, a peer-to-peer network consists of numerous connected computers called nodes. Each node in the network has a direct or indirect connection with the other network nodes. Each node makes a portion of its computational resources (ie, processing power or storage capacity) available directly to other nodes, without the need for central coordination by servers [33]. Unlike centralized networks, peer-to-peer networks have no central control, and each network node is equal to all others. Furthermore, all nodes function as both servers and clients. Figure 1 illustrates the architecture of the centralized and peer-to-peer networks.

- Distributed ledger: all transactions in the network are stored in a shared ledger. This consists of a chain of blocks, with each block containing a set of transactions. Each block is timestamped and linked to the blocks immediately preceding it. Each node maintains an identical copy of the shared ledger. To add a new transaction, the network nodes use a consensus protocol to evaluate and verify the new transaction. This protocol guarantees that a transaction is appended to the shared ledger only if most nodes validate the transaction. Once the transaction is appended to the shared ledger, it cannot be changed or reverted, and because all nodes have an identical copy of the shared ledger, no node has the power to change the data. This ensures the integrity of the shared ledger. However, recent research has proven that altering the shared ledger is feasible with 51% attacks where an adversary can control more than half of the total nodes in the blockchain network to alter the shared ledger [34]. Figure 2 illustrates a simplified blockchain concept.

##### Types of Blockchains

In terms of access to data and the role of nodes participating in the network, blockchain is classified into 4 types [35].

1. Public permissionless. Anyone can participate in the network and read or write data from the blockchain. Bitcoin and Ethereum are examples of a public permissionless blockchain.
2. Public permissioned. Anyone can participate in the network and read data from the blockchain, but a limited set of participants can write data in the blockchain. Ripple [36] and EOSIO blockchain [37] are examples of public permissioned blockchains.
3. Private permissionless. A limited set of participants can participate in a network in which all participate can read or write data from or in the blockchain. Holochain [38] is an example of a private permissionless blockchain.
4. Private permissioned. A limited set of participants can participate in the network and read data from the blockchain, but a subset of them can write data in the blockchain. Hyperledger Fabric [39] and Hyperledger Besu [40] are examples of privately permissioned blockchains.

#### Dynamic Consent and Blockchain

Dynamic consent is a two-way communication method that enables individuals to specify what data they are willing to share with various health care providers by setting and modifying their consent preferences. It enables individuals to control their data by granting and revoking access to their data, tracking their data, and updating their consent preferences. Despite these benefits, the implementation of dynamic consent in clinical genetics is limited because of ethical, legal, and data security concerns. The lack of patient trust [41,42], confidentiality data and misuse [42,43], and the lack of traceability and transparency mechanisms [44-47] are among the greatest concerns. Blockchain technology has many appealing properties, such as immutability, transparency, and accountability, that can address some of the barriers that inhibit the implementation of dynamic consent. Blockchain can support dynamic consent, as follows: data transparency and accountability through an immutable ledger, data security and privacy using cryptography mechanisms, and an efficient management system through smart contracts.

## Methods

### Blockchain Potential in Genomic Data Sharing

**Figure 3:**
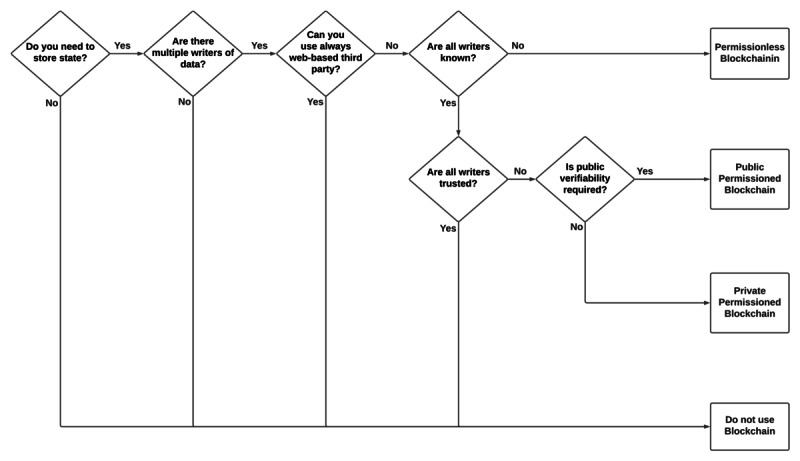
Decision tree to determine the use of blockchain [48].

Determining whether blockchain is applicable to a particular scenario is not an easy task. Although no general formula or rule exists for the applicability of blockchain, several decision schemes have been proposed to determine whether a blockchain should be used depending on situational requirements [48-50]. Wüst and Gervais [48] proposed a decision tree to identify the scenario-based applicability of blockchain, as shown in Figure 3. This decision tree consists of 6 questions. Next, we answer these questions by considering our genomic data-sharing scenario.

1. Do you need to store state? The answer to this question is yes. Diagnosing a patient with a rare genetic disease is a complex and time-consuming task, as it involves gathering data from multiple sources [51]. For instance, to answer a simple question of whether a mutation in a patient associated with a particular disease has been previously reported with the same or similar disorders in another individual requires accessing preexisting genetic and phenotypic data from multiple databases relevant to the clinical case [51,52]. Therefore, uniform access to preexisting genotype and phenotypic data using blockchain could improve the discovery and diagnosis of rare diseases. Moreover, accessing such databases involves legal and ethical obligations, including patient consent. For example, patients must control their own data and keep track of who has access to their data at any given time. Therefore, the storage and collection of patient consent as well as the administration of consent and data traceability will be guaranteed by using blockchain.
2. Are there multiple writers of data? In clinical genomics, multiple parties are involved in the patient treatment pathway, such as clinicians, scientists, and clinical laboratory technicians [51]. Therefore, a single source of truth is required for the patient data. Owing to the immutability of blockchain, the existence of patient data as well as the ownership and integrity of the data can be guaranteed. Therefore, considering that multiple parties would produce and deliver patient data, this question can be answered with yes.
3. Can you use an always web-based trusted third party? Trust and consent are important factors in the successful advancement of genome medicine and research. Patients should feel confident that their data are handled safely and are only used with their consent. A recent Genome UK report [53] showed that patients and the public are optimistic about the potential of genome medicine, but they have concerns related to the security and use of their data. It is reasonable to mention that patients trust health care providers more than any third party with their data. However, because of the high profile of patient data breaches [54,55] by health care providers, this trust has been broken. Blockchain can eliminate the need for a trusted party by establishing trust between system actors through its robust technical infrastructure and cryptography mechanisms. Therefore, the answer to this question is probably no.
4. Are all writers known? To produce, manage, and store patient data, health care providers must identify themselves. Moreover, patients need to identify themselves to connect with health care providers. Therefore, a clear answer to this question is yes.
5. Are all writers trusted? Although a minimum level of trust is required between patients and health care providers, health care providers might use patient data for research purposes without obtaining explicit consent from patients [56-58]. Blockchain enables accountability and transparency in the system by providing an audit trail and traceability of the stored data, which in turn reinforces patients’ trust in health care providers. Therefore, the answer to this question is probably no.
6. Is public verifiability required? Even though patient data are not stored in the blockchain directly (off-chain storage), access to the system should be private and permissioned. Thus, the answer to this question is no.

On the basis of the answers to these 6 questions, it is clear that the use of blockchain for the proposed genomic data sharing scenario is justifiable.

### Design Requirements

#### Overview

To identify the design requirements for ConsentChain, we analyzed a recent deliberative focus group study with National Health Service (NHS) Genomic Medicine Service patients regarding public opinion on sharing genomic data (National Research Ethics Committees ethical approval reference 18/NW/0510) [59]. We used the user stories method [60] to capture the main system design requirements. We used card sorting to collect data from the manuscript. We used our interpretation to represent the statements made by the study participants in simple user stories. We then discussed these user stories with a focus group study team to refine them. We emphasize that the findings from the focus group study are partially applicable to the scenario of our blockchain use case. Finally, 6 design requirements were identified.

#### Requirement 1: Data Discovery

##### User Stories

As a patient, I want my data to be available for sharing to facilitate my diagnosis and treatment.

As a patient, I want my unidentifiable data to be available for wider sharing to help others’ treatment and facilitate extensive research.

As a patient, I want my data to be available for different healthcare providers, so I won’t have to repeat myself every time I visit a new healthcare provider.

##### Context

The study participants allowed the sharing of their genomic data to support the diagnosis and treatment of their conditions across multiple health care providers. They also agreed to use their genomic data to benefit other patients with similar genetic conditions and for future research.

##### Implications for System Design

The system should allow information about a genomic data set of interest stored in an individual genetic laboratory to be discoverable and accessible by health care professionals and researchers.

#### Requirement 2: Data Security

##### User Stories

As a patient, I want best practices in data security to be implemented to protect my data so that it can be safeguarded against hacking and loss.

As a patient, I want to have different levels of purpose to access my data, so they can be used for authorised purposes.

##### Context

There was consensus among the participants that genomic data should be stored and shared securely without unauthorized alteration while making them available for authorized purposes.

##### Implications for System Design

Security techniques, such as data encryption and access control, should be used to protect sensitive data. Owing to the open and transparent nature of blockchains, sensitive data (either encrypted or not) should not be stored in the chain.

#### Requirement 3: Data Privacy

##### User Stories

As a patient, I want my genetic data to be shared without my identifiable information (eg, my name), so my identity will not be compromised.

##### Context

The participants emphasized that sharing genomic data outside of the patient’s direct care should be anonymized to protect their identity.

##### Implications for System Design

The system should allow the flow of patient data among involved parties while minimizing the risk of patient identity disclosure.

#### Requirement 4: Patient Control Over Data and Requirement 5: Traceability

##### User Stories

As a patient, I want to give my consent to share my data for certain purposes that are clearly outlined so that no further consent is required for these purposes.

As a patient, I want to be told whether the purpose of sharing my data is changed so I’ll have the option of giving explicit permission for the new changes.

As a patient, I want to have the option to update/withdraw my consent in a straightforward and easy way so I can change my mind later.

As a patient, I want to be able to track my shared data so that I know when and with whom my data are being shared.

##### Context

The participants thought that they should be asked for permission to share their data and be informed about how their data would be used and for what purpose. Moreover, some believed that they would exercise their right to opt out.

##### Implications for System Design

The system should enable patients to update their permissions dynamically and track data that are being shared with different parties.

#### Requirement 6: Minimum Data Disclosure

##### User Stories

As a patient, I want to have different levels of role requesters designated to access my data so only authorised parties can gain access.

As a patient, I want to have a time limit for my shared data, so they cannot be used for other purposes in the future.

##### Context

Some participants were concerned about unauthorized disclosure of their data to third parties, including family members, employers, and law enforcement agencies, whereas others were concerned with restricting access to their data by commercial entities.

##### Implications for System Design

The system should be designed in a way that allows the sharing of patient data for a given time frame and specific purpose.

### Consent Elements

**Figure 4:**
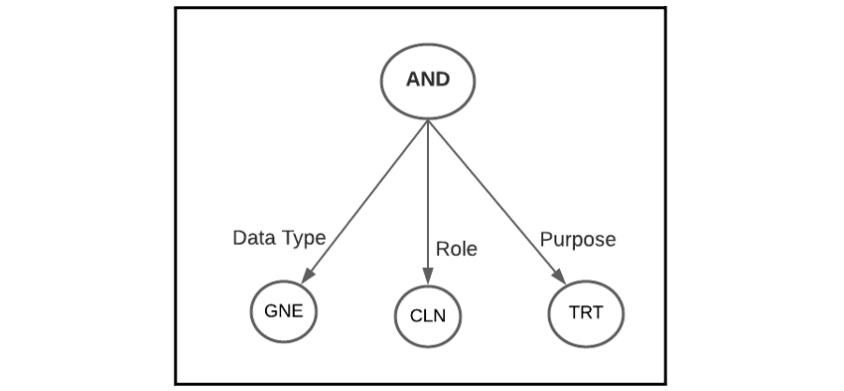
Example of an access policy tree where patient genotype data to be accessed by a clinician for treatment. CLN: clinician; GNE: patient genotype data; TRT: treatment.

Inspired by the Global Alliance for Genomics and Health (GA4GH) data use ontology effort to model genomic data use restrictions and data access requests [61,62], we developed an ontology model to represent patient consent elements into machine-readable codes. The model includes consent elements describing the data type, purpose, and role of the data requester (DR). Tables 1-3 show an abstract view of the consent elements and their codes. We also introduced an access policy tree representing a Boolean formula that defines a combination of consent elements. Any data access request that satisfies the tree can obtain access to patient data. Figure 4 shows an example of an access policy tree that allows patient genotype data to be accessed by a clinician for treatment.

**Table 1:** Code representing the data type in consent element.

Data type	Code
Genotype	GNE
Phenotype	PHE
Metadata	MEA

**Table 2:** Code representing the role in consent element.

Purpose	Code
Treatment	TRT
Research	REH
Clinical	CLL

**Table 3:** Code representing the purpose in consent element.

Role	Code
Clinician	CLN
Researcher	REE
Bioinformatician	BIN

### Related Work

**Figure 5:**
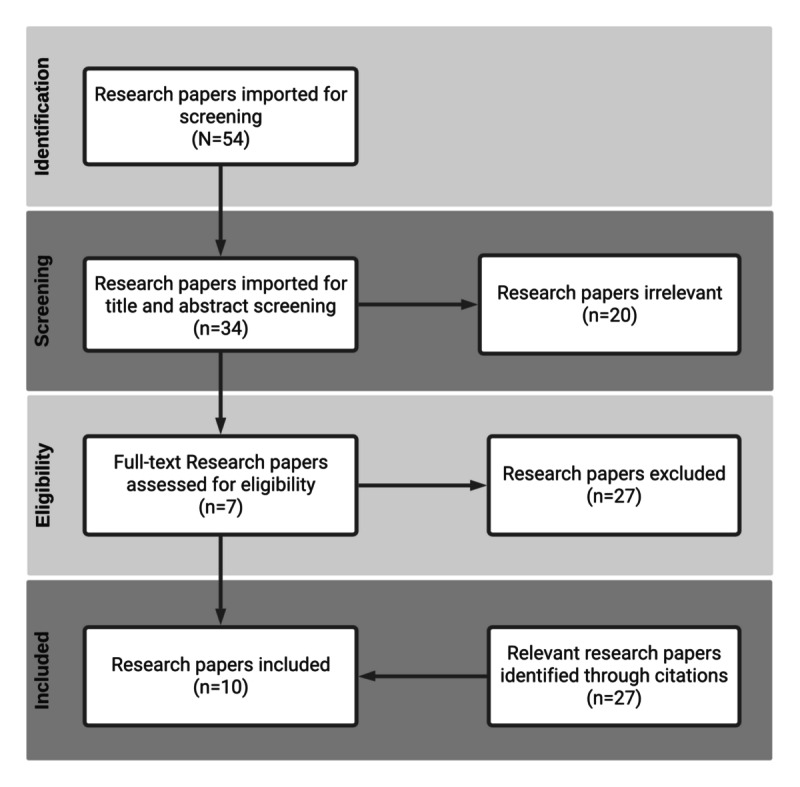
PRISMA (Preferred Reporting Items for Systematic Reviews and Meta-Analyses) flow for this review.

We used PRISMA (Preferred Reporting Items for Systematic Reviews and Meta-Analyses) guidelines to conduct a systematic review to analyze the existing literature on blockchain-based consent data used in health care management systems. The PRISMA flowchart for this systematic review is shown in Figure 5. For the purposes of this review, a reputable database (PubMed) was searched using the search query shown in Textbox 1. The resulting research papers (N=54) were imported into Covidence, a web-based app tool used to manage systematic reviews. In the next step, research papers were screened against titles and abstracts, and research papers unrelated to consent management systems were excluded (n=20). Then, the remaining research papers (n=34) were assessed for full-text eligibility, with the following exclusion criteria:

- No consent management explained (n=13)
- No implementation provided (n=2)
- No access to the full text (n=2)
- Reviews and ideas (n=6)

**Research query.**

((blockchain[Title/Abstract]) OR (Smart contracts [Title/Abstract]) OR (blockchain-based[Title/Abstract]) OR (Smart contracts-based[Title/Abstract])) AND ((Consent*[Title/Abstract]) OR (permission*[Title/Abstract]) OR (access control[Title/Abstract])) AND ((healthcare[Title/Abstract]) OR (EMR[Title/Abstract]) OR (genomic[Title/Abstract]) OR (Genetic [Title/Abstract]) OR (electronic health records[Title/Abstract]) OR (EHR[Title/Abstract]) OR (electronic Medical Records [Title/Abstract]) OR (Medical[Title/Abstract]) OR (Clinical Trial[Title/Abstract]) OR (Patient*[Title/Abstract]))

Additional relevant research papers were identified through citations (n=3). The remaining research papers and the identified relevant research papers (n=10) were analyzed thoroughly. The final findings are summarized in Multimedia Appendix 1 [63-72].

Chenthara et al [63] proposed a blockchain-based privacy-preserving framework called Healthchain to support electronic health record (EHR) access control and management. The framework was implemented using the Hyperldger Fabric InterPlanetary File System (IPFS). To achieve the immutability of EHRs, they were stored off-chain in an IPFS, with only the hash values of the EHRs being stored in the blockchain. Smart contracts were used to model the logic of EHR transactions in the system, including data exchange, access management, and EHR management. Azaria et al [64] proposed a decentralized management system called MedRec, which was built using Ethereum smart contracts to facilitate the management of EHRs between health care providers. MedRec enables patients to have full control over their data by granting or revoking access to their data. To keep patients anonymous, their identification strings are mapped to their blockchain addresses. Smart contracts are used to define how data are managed and accessed. MedRec provides an immutable access history summary that improves accountability and transparency in the system. It can be integrated with current providers’ existing databases, and other medical stakeholders can participate.

Cryan [65] proposed a blockchain-based architecture capable of enabling patient data sharing across hospital systems. The proposed architecture was implemented using Ethereum smart contracts and IPFS to protect sensitive patient data and enable patients to own and share their data with designated clinicians and revoke that permission later. Choudbhury et al [66] developed a decentralized system using Hyperledger Fabric for informed consent management and secondary data sharing. The system enhances compliance in human subject regulations for institutional review board regulations by leveraging smart contracts to enable a quick and efficient recording of consent and enforce the guidelines of a clinical trial protocol. Mamo et al [67] presented a well-designed system called Dwarna that harnesses blockchain technology to enable dynamic consent in biobanking. This system aims to increase transparency by storing the research participants’ consent changes on the blockchain and presents a solution to overcome the blockchain incompatibility with Article 17 of the European Union’s General Data Protection Regulation (GDPR), known as the right to erasure, by using a different representation of research participants in both off-chain databases and blockchain. The proposed system was implemented using a Hyperledger Fabric blockchain.

Tith et al [68] proposed a blockchain-based consent management model to support the sharing of EHRs. The model was implemented using Hyperledger Fabric and where smart contracts were used to manage patient consent. Patient consent preferences, metadata of patient records, and data access logs are stored immutably on the blockchain, enabling transparency and traceability of patient data and consent. Dubovitskaya et al [69] proposed a secure blockchain-based record management system that facilitates the secure sharing and aggregation of EHR data. The system is patient-centric and allows patients to manage their own EHRs across multiple hospitals. It uses proxy re-encryption algorithms and a fine-grained access control mechanism to ensure patient privacy and confidentiality. Dubovitskaya et al [70] proposed a framework on a permissioned blockchain for sharing EHRs for care of patients with cancer. The proposed framework is implemented with the Hyperledger Fabric blockchain and uses a membership service to authenticate registered users using username or password credentials. To create patient identity, personally identifying information, such as name, social security number, and date of birth, are hashed and encrypted for security. Medical data were stored off-chain in secure cloud storage, where access management is managed by smart contract logic.

Rajput et al [71] presented a blockchain-based access control framework that maintains patient data privacy under emergency conditions. The framework was implemented on the permissioned blockchain Hyperledger Fabric, and smart contracts were used to enable patients to manage the access rules for their data. The system keeps the history-of-transactions logs while patients are in an emergency, enabling auditing at any time point. Zhuang et al [72] presented a generalized blockchain-based architecture that provides generic functions and methods for a wide spectrum of health care apps. These functions and methods include requesting patient data, data access permission granting or revoking, and data tracking. The presented architecture was implemented on the Ethereum blockchain in 2 relevant health app domains: health information exchange and subject recruitment for clinical trials.

Compared with existing relevant literature, the proposed system is dynamic and supports minimum data disclosure. To the best of our knowledge, no relevant literature has reported on the 6 design requirements and provides a detailed analysis of the system performance. Multimedia Appendix 1 [63-72] summarizes the literature for blockchain-based consent management systems.

### System Architecture

**Figure 6:**
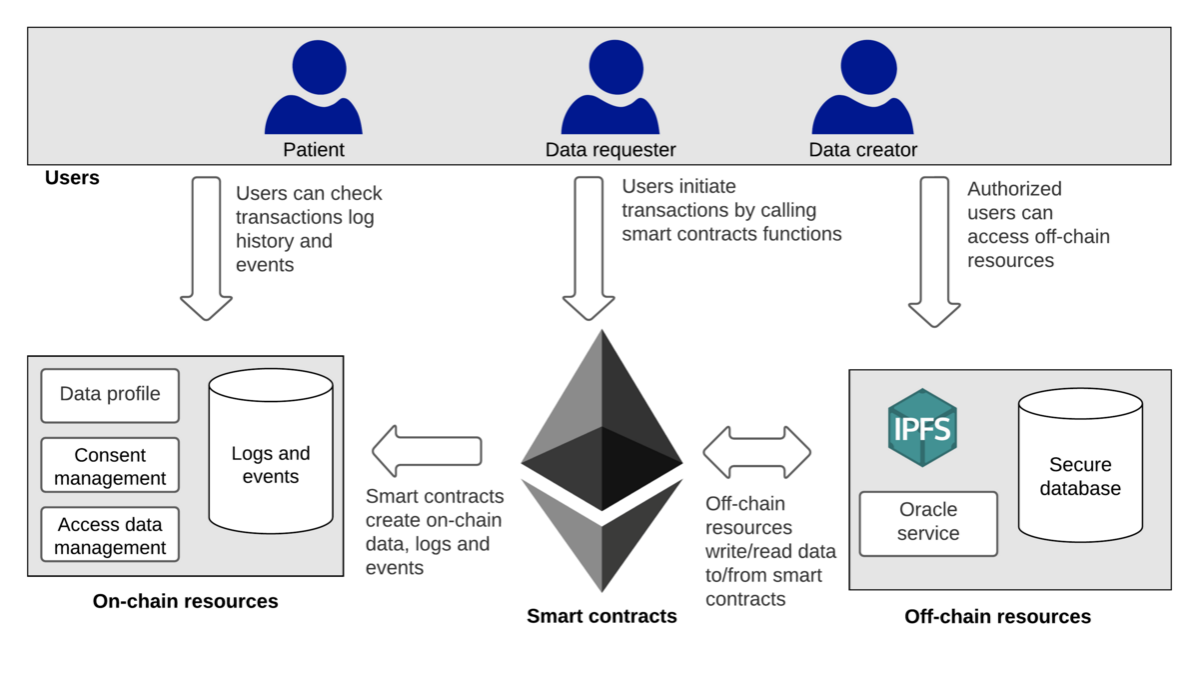
The components of the proposed architecture. IPFS: InterPlanetary File System.

In this section, we describe the proposed blockchain-based dynamic consent architecture for supporting clinical genomic data sharing. This generic architecture can be customized and used in different use cases where dynamic consent is required. As illustrated in Figure 6, the components of the proposed architecture are as follows: 

1. Users
  - A data creator (DC): an organizational entity, such as a genetic testing laboratory, where patient data are collected and stored in secure databases.
  - Patient: an individual whose data are stored off-chain in a secure database managed by the DC; a patient can provide consent to the system using the
    consent elements
    code.
  - DR: a domain expert or organizational entity that wishes to discover and request access to patient data for a specific purpose, including research and health care.
2. Smart contracts, which are used to provide system functionalities, such as registering new users, managing patient consent, and processing access requests to patient data. In addition, smart contracts create transaction logs and events that enable the tracing and auditing of all system data and actions.
3. On-chain resources
  - Logs and events: smart contracts create logs and events for all system transactions. These logs and events are stored on-chain, and they are an important resource for tracing and auditing all system actions, thus making all system users accountable for their actions.
  - Data profile (DP): This is a description of preexisting genomic data for a specific patient that is stored off-chain in a genetic laboratory database. A patient DP contains information including the location of the patient data, patient condition, and gene name, and it does not reveal any sensitive and identifiable information. Storing patient DPs on-chain helps the DR to discover and identify a genomic data set of interest stored in several genetic laboratory databases.
  - Consent management: This is used to handle patient consent operations, such as adding, updating, and deleting consent. 
  - Access data management: This is used to handle access to patient data procedures, including validating access requests and providing secure access to off-chain data.
4. Off-chain resources
  - Secure database: a private database managed by a DC in which all information related to the required DP is stored.
  - Oracle service: by design, blockchain and smart contracts cannot access and read off-chain data; therefore, oracle services are used. An oracle service is a trusted data feed service that provides off-chain data to the blockchain. In the proposed system, an oracle service is used to enable smart contracts to communicate with a secure database. 
  - IPFS: This is a decentralized file storage system that stores and shares various types of files permanently. Each stored file is given a unique hash value based on its content. This hash value is then used to retrieve the file from the system. In the context of this study, we leverage IPFS as a key management service to store users’ public key (PU). We believe that IPFS is the best candidate for users’ PU because of its high availability and low cost.

## Results

### Implementation

#### Overview

**Figure 7:**
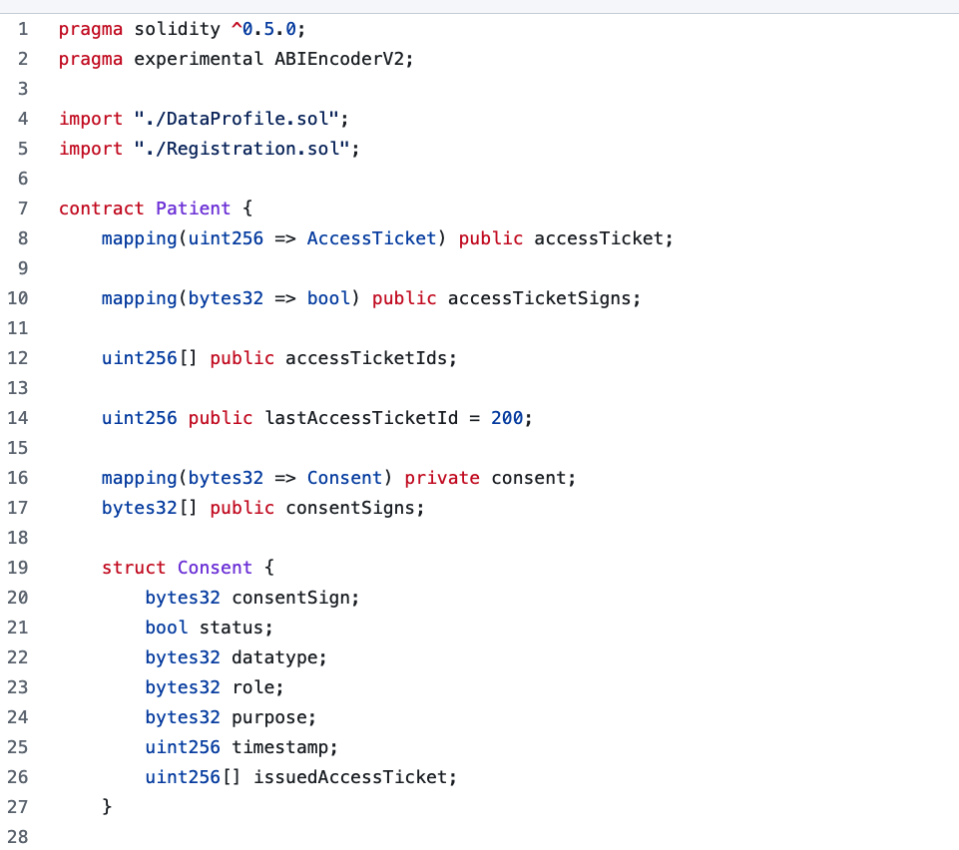
An illustrative example of patient smart contract code.

We implemented our proof-of-concept on a privately permissioned blockchain to demonstrate the feasibility of our blockchain-based architecture. At the infrastructure level, Hyperledger Besu [40], an open-source Ethereum client that provides permissioned private blockchain networks, was used to build a private blockchain. The Solidity programming language was used to write the system smart contracts and truffle framework, a development tool for developing and testing Ethereum smart contracts, to test, compile, and deploy system smart contracts. Figure 7 shows a portion of the patient’s smart contract code. Finally, we used Provable [73] as an oracle service and MongoDB to create an off-chain database.

Six smart contracts are written to manage on-chain transactions: registration smart contract (RSC), patient smart contract (PSC), data profile smart contract (DPSC), data creator smart contract (DCSC), data requester smart contract (DRSC), and oracle service smart contract (OSSC). These smart contracts provide 8 main system functions: *createNewDataRequestorContract*, *createNewPatientContract*, Cr*eateNewDataCreatorContract*, *setConsent*, *cancelConsent*, *checkConsent*, *setupDataProfile*, *requestAccessTicket*, and *requestAccessToken*. We used smart contract modifiers to restrict the calling of these functions to authorized users. Any unauthorized function call results in stopping the execution of the function and reverting all changes to the original state. The remainder of this section explains the implementation of the main system functionalities using smart contract functions.

#### Registration

Each system participant interacts with the system via his or her smart contract, which includes all the required information to interact with the system. Therefore, the participant should be registered in a system in which a smart contract is created. All users’ identities and professional registrations should be verified by a system admin, who is responsible for setting up the system and inviting the authorities to join the system, such as the NHS, before proceeding with the process of system registration. Textboxes 2-4 describe the user registration process for the patient, DC, and DR, respectively. The system admin executes a specific smart contract function for each user, which creates a new smart contract and assigns the user as the owner of the contract. This is done by using modifiers to restrict the calling of the user smart contract functions to the user’s Ethereum address.

**Pseudocode of registering new patient.**

Algorithm 1:createNewPatientContracter

Input:caller, patientWalletAddress

Output: smartContractAddress

If caller=admin∧patientWalletAddress≠null then

Create newPatientSmartContract

Set newPatientSmartContract owner to patientWalletAddress

Output newPatientSmartContract address

Else

Revert smart contract state and show an error message

**Pseudocode of registering new data creator.**

Algorithm 2:createNewDataCreatorContract

Input: caller, dataCreatorWalletAddress

Output: smartContractAddress

If caller = admin∧dataCreatorWalletAddress ≠ null then

Create new DataCreatorSmartContract

set newDataCreatorSmartContract owner to

dataCreatorWalletAddress

Output newDataCreatorSmartContract address

Else

revert smart contract state and show an error message

**Pseudocode of registering new data requester.**

Algorithm 3: createNewDataRequestorContract

Input: caller, dataRequesterWalletAddress, dataRequesterPUK

Output: smartContractAddress

If caller=admin∧dataCreatorWalletAddress≠null∧

dataRequesterPUK≠null then

Create newDataRequesterSmartContract

Set newDataRequesterSmartContract owner to

dataRequesterWalletAddress

set newDataRequesterSmartContract’s public key to dataRequesterPUK

output newDataRequesterSmartContract address

Else

revert smart contract state and show an error message

#### Consent Management

Textbox 5 describes the process of creating and storing patient consent by submitting the elements of the access policy tree, which represents the patient’s consent, to the patient’s smart contract". The tree elements are then hashed to create a consent signature, which is then stored in the patient’s smart contract. A mapping data structure, a data structure type that consists of key types and corresponding value type pairs, is used to store the consent signature, which is used as a key associated with a Boolean value to indicate its status (eg, the value is true for valid consent and false for invalid consent). Hashing and storing the consent tree in a mapping data structure would enable efficient consent status retrieval and validation. As shown in Textbox 6, if the patient wants to cancel his or her consent, the associated value with the consent signature would be set to false. Textbox 7 describes the process of checking a patient’s consent status by returning the associated value with the consent signature.

**Pseudocode of storing patient consent**

Algorithm 4: setConsent

Input: caller, dataType, role, purpose

Output: status

CONSENT←mapping

If caller=contractOwner∧dataType ≠ null ∧ role ≠ null ∧ purpose ≠ null, then

h←hash(dataType, role, purpose)

if CONSENT.contain(h,true) then

revert smart contract state and show an error message

else

CONSENT.insert(h,true)

Output true

Else

Revert smart contract state and show an error message

**Pseudocode of cancelling patient consent.**

Algorithm 5: cancelConsent

Input: caller, dataType, role, purpose

Output:status

CONSENT← mapping

If caller=contractOwner ∧ dataType ≠ null ∧ role ≠ null ∧ purpose ≠ null, then

h←hash(dataType, role, purpose)

if CONSENT.contain(h,false) then

revert smart contract state and show an error message

Else

CONSENT.insert(h,false)

output true

Else

Revert smart contract state and show an error message

**Pseudocode of checking patient consent.**

Algorithm 6: checkConsent

Input: dataType, role, purpose

Output: status

CONSENT←mapping

If dataType ≠ null ∧ role ≠ null ∧ purpose ≠ null, then

h←hash(dataType, role, purpose)

r←CONSENT.return(h)

output r

Else

revert smart contract state and show an error message

#### Patient Data

Textbox 8 describes the process of submitting the patient data to the system. After collecting and storing patient data in a secure, off-chain database (eg, a genomic laboratory database), the DC submits the patient metadata, a description of the patient data that does not reveal sensitive and identifiable information, such as the hash of the stored data, conditions, data type, and gene name, to the system. The patient metadata are then stored in a data structure, where the hash of the stored data is used as a key and the remaining patient data are the value. 

**Pseudocode of creating patient data profile.**

Algorithm 7: setupDataProfile

Input: caller, patientSmartContract, dataHash, condition, dataType,gene

Output: id

DATAPROFILE←mapping

i←counter

if caller = dataCreatorSmartContract ∧ patientSmartContract ≠ null ∧ datatHash ≠ null ∧ condition ≠ null ∧ dataType ≠ null ∧ gene ≠ null

then

i++

DATAPROFILE.insert(i,[patientSmartContract, dataHash, condition, dataType, gene, dataCreatorSmartContract])

output i

Else

revert smart contract state and show an error message

#### Access Management

To access patient data, the DR needs to obtain an access ticket (ATi) and access token (ATo). The ATi is used to control access to patient data, whereas the ATo is used to minimize access to the requested data to the lowest level. Textbox 9 describes the process of requesting an ATi for the patient data. After identifying a potential patient’s data, the DR must submit an ATi request to the system to provide the hash of the requested data, his role, and the purpose of accessing the data. Then, the request is verified by the patient’s smart contract in which the patient’s consent is stored. If there is valid consent that matches a DR request, an ATi is created automatically for the DR. 

**Pseudocode for requesting access tickets to access off-chain patient data.**

Algorithm 8: requestAccessTicket

Input: caller, dataProfileId, role, purpose

Output:ticketId

DATAPROFILE←mapping

If caller=contractOwner ∧ dataProfileId ≠ null ∧ role≠ null ∧ purpose ≠ null, then

d←DATAPROFILE.return(dataProfileId)

patient←d.patientSmartContract

dataType←d.dataType

h←hash(dataType, role, purpose)

if patient.CONSENT.return(h)=true then

ticket←patient.CreateAccessTicket(caller, dataProfileId)

ticket.status=true

output ticket.id

Else

revert smart contract state and show an error message

Else

revert smart contract state and show an error message

To obtain an ATo, the DR must submit a valid ATi to the system. Textbox 10 describes the process of requesting an ATo. If the ATi is still valid and patient consent has not been updated or cancelled, an ATo is generated automatically by the DC for the DR. The ATo includes a secure one-time URL that can be used to gain access to the patient data stored off-chain.

**Requesting an access token to retrieve off-chain patient data.**

Algorithm 9: requestAccessToken

Input:caller, dataProfileId, ticketId

Output: tokenId

DATAPROFILE←mapping

If caller=contractOwner ∧ dataProfileId ≠ null ∧ ticketId ≠ null, then

d←DATAPROFILE.return(dataProfileId)

dataCreator←d.dataCreatorSmartContract

patient←d.patientSmartContract

if patient.ticket[ticketId].status=true then

token

←dataCreator.createAccessToken(caller, dataProfileId)

Token.status=true

Output token.id

Else

revert smart contract state and show an error message

Else

revert smart contract state and show an error message

### A Proof-of-Concept (ConsentChain)

**Figure 8:**
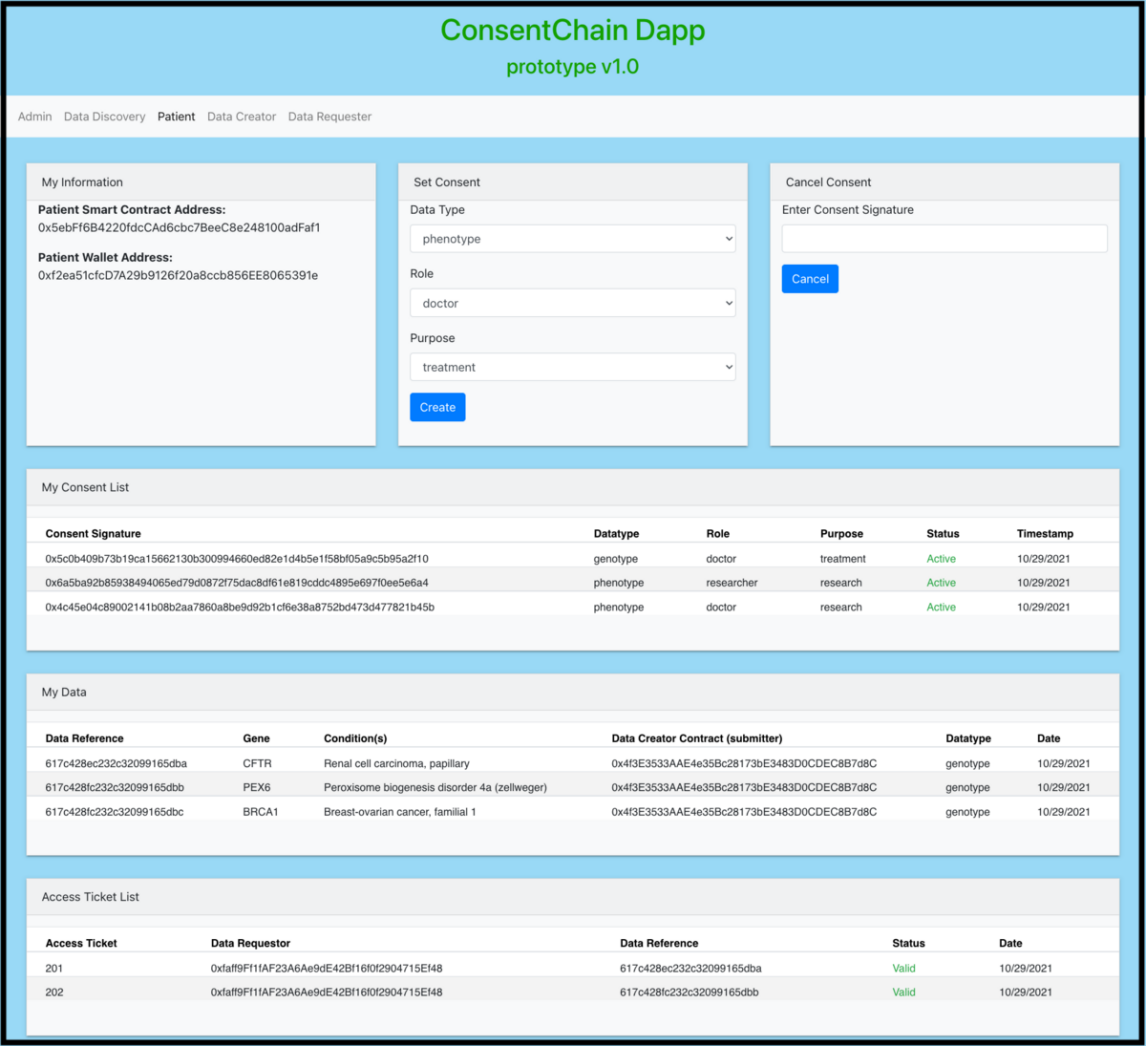
Patient interface.

**Figure 9:**
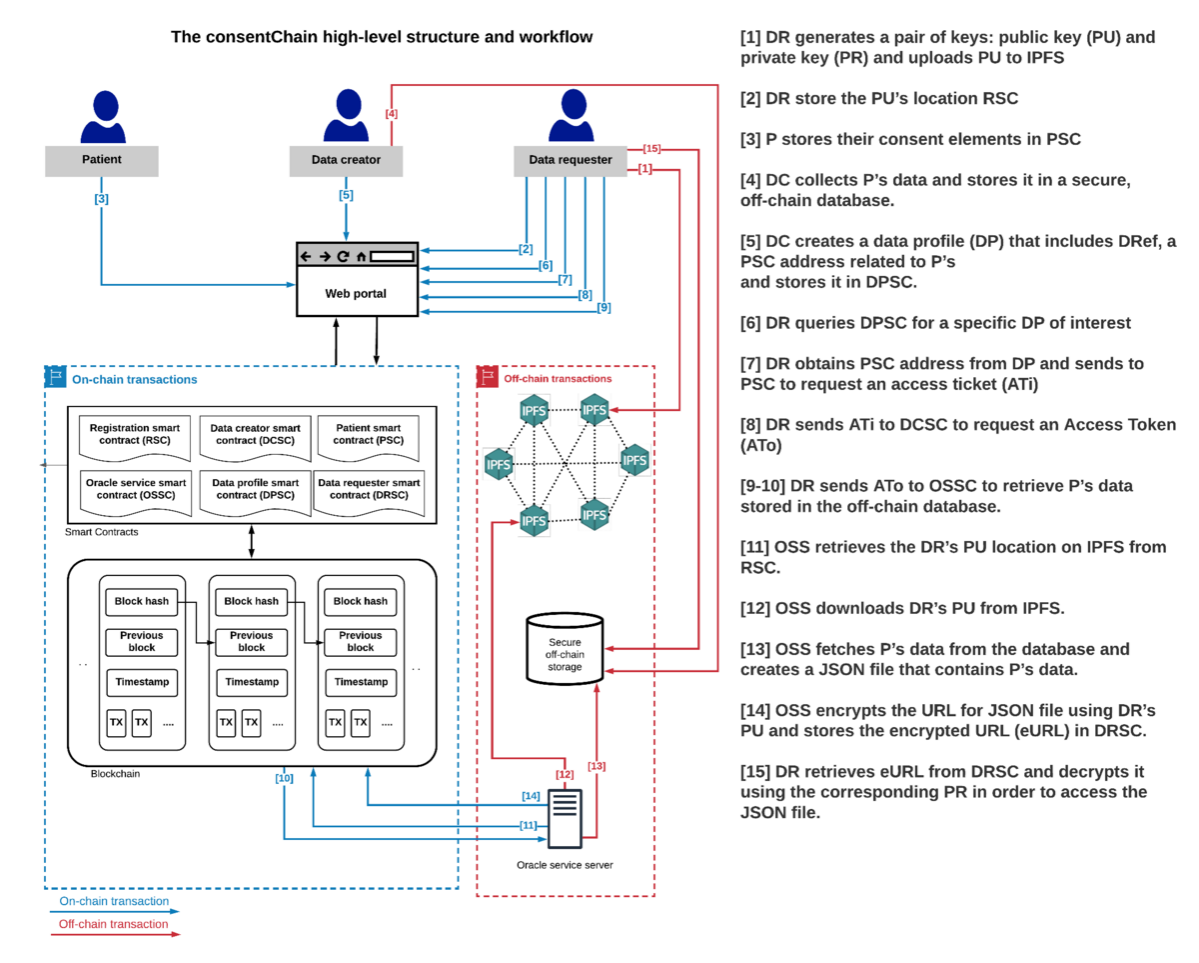
The high-level structure and workflow of ConsentChain. Ati: access ticket; DR: data requester; IPFS: InterPlanetary File System; OSS: Oracle Service Server; OSSC: oracle service smart contract; P: patient; PSC: patient smart contract; RSC: registration smart contract.

This section presents ConsentChain, a proof-of-concept implementation of the proposed architecture, to explore the efficacy of applying blockchain technology to support clinical genomic data sharing. The ConsentChain provides a web portal for patients, DCs, and DRs to interact with the system. It enables patients to provide or withdraw their consent regarding the sharing of their data and DCs to collect and store patient data and DRs to query and access patient data. Figure 8 shows the patient interface provided by the ConsentChain. The high-level structure and workflow of ConsentChain is shown in Figure 9, and the corresponding description of each step is as follows:

1. During registration, DR generates a pair of keys: a PU and a private key (PR). DR then uploads PU to the IPFS and records its location returned by the IPFS.
2. DR sends a blockchain transaction to store the PU’s location returned by the IPFS in the RSC.
3. Patient sends a blockchain transaction to store their consent elements (data type, role, and purpose) in PSC.
4. DC collects patient’s data and stores it in a secure, off-chain database. The DC also records patient’s data reference (DRef) returned by the database.
5. DC creates a DP that includes DRef, a PSC address, and other information related to patient’s data that do not reveal any sensitive and identifiable information. Then, the DC sends a blockchain transaction to store the DP in the DPSC.
6. DR queries DPSC to discover a specific DP of interest and reads transaction information related to that DP.
7. DR obtains the PSC address from the DP and sends a blockchain transaction to the PSC to request an ATi to access patient’s data stored in the off-chain database. The request is accepted or rejected automatically, based on patient consent stored in the PSC. On acceptance, ATi is generated and stored in PSC, and DR receives the transaction ID related to ATi.
8. DR sends a blockchain transaction including ATi to DCSC to request an ATo to retrieve patient’s data stored in the off-chain database. The request is accepted or rejected automatically based on ATi validation. On acceptance of the request, the ATo is stored in the DCSC, and DR receives the transaction ID related to the ATo.
9. DR sends a blockchain transaction including ATo to the oracle service smart contract to retrieve patient’s data stored in the off-chain database. The request is accepted or rejected automatically based on the ATo validation.
10. On acceptance of the request, the request is forwarded to the Oracle Service Server (OSS).
11. OSS retrieves the DR’s PU location on the IPFS from the RSC.
12. OSS downloads the PU of the DR from the IPFS.
13. OSS fetches patient’s data from the database and creates a temporary JSON file that contains patient’s data. This JSON file can be accessed via HTTPS requests and is available for one-time access.
14. The OSS encrypts the URL for a JSON file using the PU of the DR. Then, the OSS sends a blockchain transaction to store the encrypted URL in the DRSC.
15. DR retrieves encrypted URL from DRSC and decrypts it using the corresponding PR to access the JSON file.

## Discussion

### Principal Findings

In this section, we discuss how our proof-of-concept, ConsentChain, meets the requirements captured from the patient forum, and we provide a detailed analysis of its performance.

### Addressing Requirement

#### Requirement 1: Data Security

In ConsentChain, we used a hybrid data storage model that included on-chain or off-chain storage. Sensitive patient data are stored securely off-chain, whereas metadata for patient data are stored on-chain along with a reference pointer to the data source. This reference pointer is constrained by a short time frame and is encrypted. Only an authorized DR can decrypt it within the given time frame to access patient data. Moreover, implementing ConsentChain on a private or consortium blockchain adds a security layer in which all users are verified before joining the network.

#### Requirement 2: User Control Over Data

Smart contracts act as autonomous actors whose behavior is predictable [74]. However, because of blockchain immutability, once a smart contract is deployed, it cannot be modified; hence, bugs and security vulnerabilities found in the deployed smart contract are difficult to resolve. Therefore, smart contract security audits and testing are essential for developing smart contracts to minimize the risk of mismatches between a smart contract intended behavior and the actual behavior [75]. Using a smart contract to manage consent would enable patients to dynamically grant and revoke access to their data. In ConsentChain, patients record consent preferences in their smart contract, and they can amend or delete these preferences at any time. These changes were reflected in the system in real time.

#### Requirement 3: Data Privacy

By leveraging blockchain authenticity and verifiability features, ConsentChain maintains privacy by using permissioned blockchain and anonymized accounts. Only authorized users can access the blockchain via their anonymized accounts, enabling patients to provide their consent without revealing their real identities.

#### Requirements 4 and 5: Data Discovery and Minimum Data Disclosure

In the health care context, balancing the maximization of data discovery while minimizing data disclosure risk is a challenging task [76-78]. Inspired by the one-time password scheme, we proposed a one-time-access-token mechanism to minimize the data disclosure risk in ConsentChain. In this mechanism, an ATo is automatically generated for an authorized access request. The token is valid for one-time use, and it contains an encrypted reference pointer to the data source along with a digital signature on the shared data to ensure data integrity against tampering. Only an authorized DR can decrypt the reference pointer to access the data within a given time frame. If the DR needs to access data in the future, the generation of a new ATo is required. Through the implementation of a one-time access-based token and public-key cryptography, a compromised reference pointer to patient data will not lead to data leakage. This is because of the limited access and time restrictions given to access patient data, further increasing the security of ConsentChain and decreasing the likelihood of data leakage.

To maximize data discovery, we leveraged the blockchain features. One of these is the replication of data stored on-chain across the network; a consensus mechanism ensures that each node obtains a local identical copy of the data. Using their local copy of the on-chain data, a DR can identify potential patient data instead of individually querying each off-chain storage. Therefore, storing patients’ metadata on the chain would provide DRs with a broader vision of similar patient data, which are stored off-chain across different laboratories.

#### Requirement 6: Traceability

By leveraging the blockchain’s immutability, our system maintains an immutable log of all system transactions. As the process of sharing patient data is managed by smart contracts, all involved transactions are recorded permanently on the blockchain. This would enable patients to inspect the blockchain for all information and transactions related to their data, including where data are stored off-chain and who have access to them and for what purpose. This feature is a significant upgrade toward patient-centric approaches to advance data sharing. It would also enable regulators to investigate claims in the event of disputes among involved parties, thereby increasing confidence in ConsentChain.

### Security Analysis

This section provides a security analysis of ConsentChain in terms of patient privacy preservation, data storage, data sharing, and tamper-proofing.

#### Patient Privacy Preservation

Genomic data are highly sensitive and should not be disclosed without proper permission. In ConsentChain, genomic data are stored in an off-chain private secure storage with an access control mechanism, thereby reducing the risk of patient data leakage. Moreover, to ensure participant anonymity, a randomly generated unique account was generated for the participants who were associated with a PU. This account is used to send transactions to the blockchain; these transactions are anonymous and cannot be linked to the real identity of participants. In addition, multiple accounts can be created for one participant; hence, transactions sent to the blockchain by the same participant cannot be inferred by an adversary.

#### Data Storage

In ConsentChain, genomic data are stored in an off-chain private secure storage system. The security of this storage is beyond the scope of this paper, and we assume that it is secured by its owner (the DC). Only the metadata, hash, and reference of the off-chain stored data are shared on the blockchain. The off-chain DRef stored in the blockchain is tamper-proof.

#### Data Sharing

Only authorized users can request access to off-chain data through permissions that are preset in smart contracts. After receiving a valid request, the DC creates a JSON file that contains the requested data and stores it in the temporary access off-chain storage from where it can be accessed via HTTPS. Access to the JSON file is restricted by a one-time visit and a short time frame. The DC then retrieves the PU of the user who requested the data from the IPFS and encrypts the URL that allows access to the JSON file and then stores it in the blockchain. The user requesting the data can then obtain the URL from the blockchain and decrypt it using their PR and access the JSON file. Once the JSON file is accessed, it is removed from the temporary access off-chain storage, making the URL stored in the blockchain useless; therefore, if the adversary compromises the PR of the user requesting the data to decrypt the URL, the URL would lead to nothing. Further, if the JSON file is not accessed within the specified time frame, it is removed from the temporary access off-chain storage, reducing the risk of unauthorized access to the data.

#### Tamper-Proofing

In ConsentChain, data access activities are recorded in the blockchain and can be audited and tracked. In addition, the data stored in the blockchain are immutable and cannot be arbitrarily modified owing to the consensus mechanisms used in the blockchain, which guarantees that the added blocks cannot be modified unless an adversary can launch a 51% attack. It is worth noting that the mechanism of launching a 51% attack differs depending on the type of consensus mechanism used in the blockchain. For instance, public blockchains such as Ethereum and Bitcoin use the proof-of-work consensus mechanism, which requires high computational power to generate new blocks, whereas in a private permissioned blockchain, the proof-of-authority consensus mechanism can be used to generate new blocks [79-82]. To launch a 51% attack on a blockchain that uses the proof-of-work consensus mechanism, an adversary needs to obtain 51% of the network’s computational power. In contrast, when the proof-of-authority consensus mechanism is used, a 51% attack can only be launched by controlling over 51% of the network nodes, which is much more difficult than obtaining 51% of the network computational power [80]. Therefore, in ConsentChain, the proof-of-authority consensus mechanism is used to reduce the risk of a 51% attack.

### Performance Evaluation

To test and validate ConsentChain, we built a real production environment for the deployment and hosting of ConsentChain. A detailed performance analysis of ConsentChain is provided in Multimedia Appendix 2. In summary, the analysis of the performance of the *Transaction* and *Read* operations of ConsentChain indicated an average *Transaction Throughput* of 13.59 tps and an average *Read Throughput* of 135.78 tps. The *Transaction Latency* was 2.76 seconds, whereas the average *Read Latency* was 0.288 seconds. In addition, the system performance analysis shows that a large number of read operations (reading a state from blockchain), that is, 10,000 transactions, could be handled by the system at very low latency, whereas transaction operations are processed with higher latency owing to the complexity involved (reading or writing a state from or to blockchain).

### Conclusions

Genomic data are useful when shared within the clinical genomics community and compared with other patient data, indicating that clinicians might need to share data to efficiently treat patients. However, many challenges hinder large-scale genomic data sharing, such as the availability, discoverability, and accessibility of genomic data [8,51,52], preventing clinicians and researchers from generating an integrated view of rare genetic diseases. In this study, we proposed a blockchain-based dynamic consent architecture to support genomic data sharing and implemented a proof-of-concept for the architecture. We also developed an ontology model to represent patient consent elements into machine-readable codes to automate the consent and data access processes. The proof-of-concept has been implemented on a private Ethereum blockchain, and it shows that the proposed architecture can achieve a large-scale sharing of genomic data among the parties involved. The evaluation showed that patients achieved greater control over their data using this system. Performance analysis showed that the system was efficient and scalable.

Nonetheless, several limitations of this study need to be addressed. Owing to the openness and distributed nature of blockchain technology, verifying user identity is challenging. Our system operates under the assumption that the system is implemented on a private blockchain, and all users are invited to join the system. User identity verification is performed before one can join the system, and each user is given a pseudonymous identifier to represent them on the system. A more reliable and practical solution to overcome this issue might be linking patient identity with an external trusted source of information, such as GOV.UK Verify and NHS Identity. In addition, DR and DC identity verification could be achieved by linking to their professional registration.

Another issue is blockchain’s GDPR compliance, which needs to be considered [83-85]. Although blockchains can help dynamic consent systems comply with some GDPR objectives, including the rights to be informed and to withdraw, blockchains’ immutability seems to conflict with the GDPR, which encourages data minimization and gives data owners the right to erasure. A study conducted by the European Parliamentary Research Service concluded that although private and permissioned blockchains could easily comply with GDPR requirements, it is difficult to determine whether blockchains are, as a whole, either completely compliant or incompliant with GDPR [86]. However, since the GDPR came into effect, several studies have taken initial steps toward designing and building GDPR-compliant blockchain-based use cases [44,87-91]. Therefore, GDPR compliance should be considered during the design of blockchain-based systems [92,93].

The objective of this work was not to design a system that could be used in practice in health care environments, but to show that blockchain technology has the potential to address several genomic data sharing challenges. We found that facilitating genomic data sharing through blockchain technology and smart contracts is promising. However, they are not the complete answer, and a number of issues still need to be addressed before such systems can be deployed in practice, particularly in relation to verifying user credentials.

## Appendix

Multimedia Appendix 1 System design requirements in existing blockchain solutions in health care.

Multimedia Appendix 2 Detailed performance analysis of the proposed model.

## Abbreviations

ATi: access ticket
ATo: access token
DC: data creator
DCSC: data creator smart contract
DP: data profile
DPSC: data profile smart contract
DR: data requester
DRef: data reference
DRSC: data requester smart contract
GDPR: General Data Protection Regulation
IPFS: InterPlanetary File System
NHS: National Health Service
OSS: Oracle Service Server
PR: private key
PRISMA: Preferred Reporting Items for Systematic Reviews and Meta-Analyses
PSC: patient smart contract
PU: public key
