# Supplementary material for: A Blockchain-Based Dynamic Consent Architecture to Support Clinical Genomic Data Sharing (ConsentChain): Proof-of-Concept Study
Source: JMIR Med Inform. 2021 Nov 3;9(11):e27816. doi: 10.2196/27816 (PMC8600428; doi:10.2196/27816)
Supplement: Multimedia Appendix 1 [file medinform_v9i11e27816_app1.docx]

## Multimedia Appendix 1

**Table 1.** System design requirements in existing blockchain solutions in health care.

| Study | System design requirements | | | | | | Use case | Blockchain type | Technology | Data storage | Consent process | Consent type | Evaluation |
| --- | --- | --- | --- | --- | --- | --- | --- | --- | --- | --- | --- | --- | --- |
|  | 1^a^ | 2^b^ | 3^c^ | 4^d^ | 5^e^ | 6^f^ |  |  |  |  |  |  |  |
|  | | | | | | | | | | | | | |
| Azaria et al [64] | Yes | Yes | Yes | No | Yes | No | EMRs^g^ | Permissioned | Ethereum | Off-chain | Upon-request | Dynamic | No |
| Cyran et al [65] | Yes | Yes | —^h^ | No | Yes | No | EHRs^i^ | Consortium | Ethereum | Off-chain | Not Specified | Dynamic | No |
| Choudhary et al [66] | — | — | — | No | Yes | No | Clinical trial | Permissioned | Hyperledger Fabric | On-chain | Upon-request | Dynamic | No |
| Mamo et al [67] | Yes | Yes | Yes | No | Yes | No | Biobanking | Permissioned | Hyperledger Fabric | Mixed | Upon-request | Dynamic | No |
| Tith et al [68] | Yes | Yes | Yes | No | Yes | No | EHRs | Consortium | Hyperledger Fabric | Off-chain | Automated | Dynamic | No |
| Dubovitskaya et al [69] | Yes | Yes | Yes | No | Yes | No | EHRs | Permissioned | Hyperledger Fabric | Mixed | Upon-request | Dynamic | No |
| Dubovitskaya et al [70] | Yes | Yes | Yes | No | Yes | No | EHRs | Permissioned | Hyperledger Fabric | Off-chain | Automated | Grant only | No |
| Zhuang et al [72] | Yes | Yes | Yes | No | — | No | HIE^j^ and clinical trials | Permissioned | Ethereum | Mixed | Upon-request | Dynamic | Yes |
| Chenthara et al [63] | Yes | Yes | Yes | No | Yes | No | EMRs | Permissioned | Hyperledger Fabric | Mixed | Automated | Dynamic | Yes |
| Rajput et al [71] | Yes | Yes | Yes | No | Yes | No | PHRs^k^ | Permissioned | Hyperledger Fabric | Off-chain | Automated | Grant only | Yes |
| This study (ConsentChain) | Yes | Yes | Yes | Yes | Yes | Yes | Genomic data sharing | Private | Ethereum | Mixed | Automated | Dynamic | Yes |

^a^Data security.

^b^Data privacy.

^c^Traceability.

^d^Data discovery.

^e^User control over data.

^f^Minimum data disclosure.

^g^EMR: electronic medical record.

^h^Not available.

^i^EHR: electronic health record.

^j^HIE: health information exchange.

^k^PHR: personal health record.
