# Supplementary material for: A Blockchain-Based Dynamic Consent Architecture to Support Clinical Genomic Data Sharing (ConsentChain): Proof-of-Concept Study
Source: JMIR Med Inform. 2021 Nov 3;9(11):e27816. doi: 10.2196/27816 (PMC8600428; doi:10.2196/27816)
Supplement: Multimedia Appendix 2 [file medinform_v9i11e27816_app2.docx]

## Multimedia Appendix 2

### Performance Analysis

To test and validate our system in a real production environment, we used four VMs running on Google Cloud to build a private blockchain using Hyperledger Besu, an open-source Ethereum client that provides permissioned private blockchain networks, for the deployment and hosting of the system. We used Hyperledger Caliper [1], an open-source benchmarking tool for investigating the performance of blockchain applications, to perform multiple tests to validate system performance. Table 5 illustrates the configurations of Hyperledger Besu and the testing environment.

| Table 5 Configurations of Hyperledger Besu and the testing environment | |
| --- | --- |
| Factor | Setting |
| Nodes | Four VMs running on Google Cloud, where each VM has a 2 GHz, 4-core Intel 7 CPU |
| Peer-to-Peer Network | Hyperledger Besu v1.4.1  1 validator node  3 peer nodes |
| Consensus Protocol | Clique |
| Smart Contracts Programming Language | Solidity |
| Benchmarking Tool | Hyperledger Caliper v0.4.1 |

On the basis of blockchain technology, there are mainly two types of operations that involve invoking smart contracts functions: Transaction and Read operations. A Transaction operation involves changing a state in a blockchain from one value to another (e.g. storing new data on a blockchain or updating existing data on it). A Read operation includes operations that do no change to a state in a blockchain (e.g. fetching data from a blockchain). In this regard, we used five performance metrics to evaluate our system: *Transaction Throughput*, *Read Throughput*, *Transaction Latency*, *Read Latency and Scalability*.

*Transaction Throughput*

This metric measures the rate, in transactions per second (tps), of valid transactions submitted to the blockchain network within a defined period [2]. Equation (1) presents the Transaction Throughput.

$$\begin{matrix} Transaction Throughput= \frac{Total Committed Transactions}{Total Time \left( in seconds \right)} & \left( 1 \right) \end{matrix}$$

*Read Throughput*

This metric measures the rate, in reads per second (rps), of valid read operations completed within a defined period [66]. Equation (2) presents the Read Throughput.

$$\begin{matrix} Read Throughput= \frac{Total Read Operations}{Total Time \left( in seconds \right)} & \left( 2 \right) \end{matrix}$$

*Transaction Latency*

This metric measures the amount of time, in seconds, needed for a transaction to be executed across a blockchain network—starting from the submission of the transaction up to its addition to the blockchain [2]. Transaction Latency is expressed in Equation (3):

$$\begin{matrix} Transaction Latency= \left( Confirmation Time\times Network Threshold Time \right)-Submission Time & \left( 3 \right) \end{matrix}$$

*Read Latency*

This metric measures the interval, in seconds, between submission of a read operation and receipt of a confirmation response [2]. Equation (4) presents the Read Latency.

$$\begin{matrix} Read Throughput= Time of Reply Receipt - Submission Time & \left( 4 \right) \end{matrix}$$

*Scalability*

This metric measures the impact of the increasing number of users on throughput and latency in a blockchain-based systems. This can be measured using different parameters, i.e. block frequency, block size, workload type, node configuration, network size, the total number of transactions to be processed, actively participating nodes, consensus protocol and blockchain client type, etc. [3]. In this experiment, the total number of transactions was selected as a parameter to measure the system scalability, where each transaction represents an individual system user.

In this performance test, we focused on the main smart contracts functions that represent the Transaction and Read operations of the proposed system (Table 6). A test module was created for each Transaction and Read operation, and tests were performed in multiple rounds with a fixed number of transactions and different tps send rates to reduce the likelihood of errors due to system overload and network congestion. Tables 7 and 8 summarise parameters used for the Transaction and Read operations evaluation, respectively. Moreover, to understand the impact of the increasing system users on system throughput and latency, an additional experiment was conducted in which a different number of transactions were sent to the blockchain at a linear rate that gradually changes transaction during the test to identify transaction rates that affect the system performance. Table 9 summarises the parameters used for system scalability evaluation.

| Table 6 Main smart contracts functions of the system | |
| --- | --- |
| **Main Function** | **Operation Type** |
| Algorithm 1 | Transaction |
| Algorithm 2 | Transaction |
| Algorithm 3 | Transaction |
| Algorithm 4 | Transaction |
| Algorithm 5 | Transaction |
| Algorithm 6 | Read |
| Algorithm 7 | Transaction |
| Algorithm 8 | Transaction |
| Algorithm 9 | Transaction |

| \| \| Table 7 Experimental settings for Transaction operations \| \| \| \| \| \| \| \| \| \| \| \| --- \| --- \| --- \| --- \| --- \| --- \| --- \| --- \| --- \| --- \| --- \| \| **Test Number** \| **1** \| **2** \| **3** \| **4** \| **5** \| **6** \| **7** \| **8** \| **9** \| **10** \| \| **Functions Under Test** \| Transaction operations \| \| \| \| \| \| \| \| \| \| \| **Worker Number** \| 1 worker \| \| \| \| \| \| \| \| \| \| \| **Transaction Number** \| 100 Transactions \| \| \| \| \| \| \| \| \| \| \| **Type of Control Rate** \| Fixed rate \| \| \| \| \| \| \| \| \| \| \| **Send Rate (tps)** \| 5 \| 10 \| 15 \| 20 \| 25 \| 30 \| 35 \| 40 \| 45 \| 50 \| \| \| --- \| --- \| --- \| --- \| --- \| --- \| --- \| --- \| --- \| --- \| --- \| --- \| --- \| --- \| --- \| --- \| --- \| --- \| --- \| --- \| --- \| --- \| --- \| --- \| --- \| --- \| --- \| --- \| --- \| --- \| --- \| --- \| --- \| --- \| --- \| --- \| --- \| --- \| --- \| --- \| --- \| --- \| --- \| --- \| --- \| --- \| --- \| --- \| --- \| --- \| --- \| --- \| --- \| --- \| --- \| --- \| --- \| --- \| --- \| --- \| --- \| --- \| --- \| --- \| --- \| --- \| --- \| --- \| --- \| --- \| --- \| --- \| --- \| --- \| --- \| --- \| --- \| --- \|  \| \| Table 8 Experimental settings for Read operations \| \| \| \| \| \| \| \| \| \| \| \| --- \| --- \| --- \| --- \| --- \| --- \| --- \| --- \| --- \| --- \| --- \| \| **Test Number** \| **1** \| **2** \| **3** \| **4** \| **5** \| **6** \| **7** \| **8** \| **9** \| **10** \| \| **Functions Under Test** \| Read operations \| \| \| \| \| \| \| \| \| \| \| **Worker Number** \| 1 worker \| \| \| \| \| \| \| \| \| \| \| **Transaction Number** \| 100 Transactions \| \| \| \| \| \| \| \| \| \| \| **Type of Control Rate** \| Fixed rate \| \| \| \| \| \| \| \| \| \| \| **Send Rate (tps)** \| 50 \| 100 \| 150 \| 200 \| 250 \| 300 \| 350 \| 400 \| 450 \| 500 \| \| \| --- \| --- \| --- \| --- \| --- \| --- \| --- \| --- \| --- \| --- \| --- \| --- \| --- \| --- \| --- \| --- \| --- \| --- \| --- \| --- \| --- \| --- \| --- \| --- \| --- \| --- \| --- \| --- \| --- \| --- \| --- \| --- \| --- \| --- \| --- \| --- \| --- \| --- \| --- \| --- \| --- \| --- \| --- \| --- \| --- \| --- \| --- \| --- \| --- \| --- \| --- \| --- \| --- \| --- \| --- \| --- \| --- \| --- \| --- \| --- \| --- \| --- \| --- \| --- \| --- \| --- \| --- \| --- \| --- \| --- \| --- \| --- \| --- \| --- \| --- \| --- \| --- \| --- \| |
| --- | --- | --- | --- | --- | --- | --- | --- | --- | --- | --- | --- | --- | --- | --- | --- | --- | --- | --- | --- | --- | --- | --- | --- | --- | --- | --- | --- | --- | --- | --- | --- | --- | --- | --- | --- | --- | --- | --- | --- | --- | --- | --- | --- | --- | --- | --- | --- | --- | --- | --- | --- | --- | --- | --- | --- | --- | --- | --- | --- | --- | --- | --- | --- | --- | --- | --- | --- | --- | --- | --- | --- | --- | --- | --- | --- | --- | --- | --- | --- | --- | --- | --- | --- | --- | --- | --- | --- | --- | --- | --- | --- | --- | --- | --- | --- | --- | --- | --- | --- | --- | --- | --- | --- | --- | --- | --- | --- | --- | --- | --- | --- | --- | --- | --- | --- | --- | --- | --- | --- | --- | --- | --- | --- | --- | --- | --- | --- | --- | --- | --- | --- | --- | --- | --- | --- | --- | --- | --- | --- | --- | --- | --- | --- | --- | --- | --- | --- | --- | --- | --- | --- | --- | --- | --- | --- | --- |

| Table 9 Parameters for system scalability evaluation | | | | | | | | | | | |
| --- | --- | --- | --- | --- | --- | --- | --- | --- | --- | --- | --- |
| **Test Round** | **1** | **2** | | **3** | | **4** | | **5** | | **6** | |
| **Functions Under Test** | Transaction | | | | | Read | | | | | |
| **Worker Number** | 1 worker | | | | | | | | | | |
| **Transaction Number** | 100 | | 1,000 | | 10,000 | | 100 | | 1,000 | | 10,000 |
| **Type of Control Rate** | Linear rate | | | | | | | | | | |
| **Send Rate (tps)** | From 50 tps to 500 tps | | | | | | | | | | |

Tables 10 and 11 summarise the results of tests on the Transaction and Read operations, respectively. We measured the Transaction Throughput by changing the send rate from 5 to 50 tps within 10 testing rounds. As Figure 4 illustrates, the maximum Transaction Throughput achieved was 17.34 tps at a send rate of 45.5 tps, while the minimum was 4.73 tps at a send rate of 5.10 tps. Likewise, we measured the Read Throughput by changing the send rate from 50 to 500 tps within 10 testing rounds. As Figure 5 indicates, the maximum Read Throughput generated was 177.80 tps at a send rate of 406.50 tps, while the minimum was 46.25 tps at a send rate of 50.60 tps.

| Table 10 Results of testing on Transaction operations | | | | | | | | | | |
| --- | --- | --- | --- | --- | --- | --- | --- | --- | --- | --- |
| Test Number | 1 | 2 | 3 | 4 | 5 | 6 | 7 | 8 | 9 | 10 |
| Success | 100 | 100 | 100 | 100 | 100 | 100 | 100 | 100 | 100 | 100 |
| Failure | 0 | 0 | 0 | 0 | 0 | 0 | 0 | 0 | 0 | 0 |
| Send Rate (tps) | 5.10 | 10.10 | 15.16 | 20.20 | 25.29 | 30.30 | 35.34 | 40.41 | 45.50 | 50.53 |
| Max Latency (s) | 1.82 | 2.92 | 4.08 | 4.70 | 5.05 | 5.43 | 5.64 | 5.89 | 6.09 | 6.28 |
| Min Latency (s) | 0.38 | 0.38 | 0.38 | 0.58 | 0.50 | 0.64 | 0.74 | 0.90 | 0.76 | 0.83 |
| Avg Latency (s) | 1.13 | 1.76 | 2.34 | 2.59 | 2.87 | 3.12 | 3.24 | 3.45 | 3.51 | 3.60 |
| Throughput (tps) | 4.73 | 8.34 | 10.69 | 12.96 | 14.90 | 15.68 | 17.16 | 17.14 | 17.34 | 16.94 |

| Table 11 Results of testing on Read operations | | | | | | | | | | |
| --- | --- | --- | --- | --- | --- | --- | --- | --- | --- | --- |
| Test Number | 1 | 2 | 3 | 4 | 5 | 6 | 7 | 8 | 9 | 10 |
| Success | 100 | 100 | 100 | 100 | 100 | 100 | 100 | 100 | 100 | 100 |
| Failure | 0 | 0 | 0 | 0 | 0 | 0 | 0 | 0 | 0 | 0 |
| Send Rate (tps) | 50.60 | 101.10 | 151.60 | 203.05 | 253.50 | 305.35 | 355.90 | 406.50 | 455.55 | 508.90 |
| Max Latency (s) | 0.20 | 0.27 | 0.30 | 0.35 | 0.34 | 0.41 | 0.37 | 0.38 | 0.55 | 0.68 |
| Min Latency (s) | 0.19 | 0.19 | 0.19 | 0.19 | 0.19 | 0.19 | 0.19 | 0.19 | 0.18 | 0.19 |
| Avg Latency (s) | 0.19 | 0.19 | 0.20 | 0.25 | 0.25 | 0.30 | 0.28 | 0.29 | 0.42 | 0.53 |
| Throughput (tps) | 46.25 | 85.10 | 118.10 | 146.10 | 170.35 | 160.90 | 176.85 | 177.80 | 142.75 | 133.60 |

| 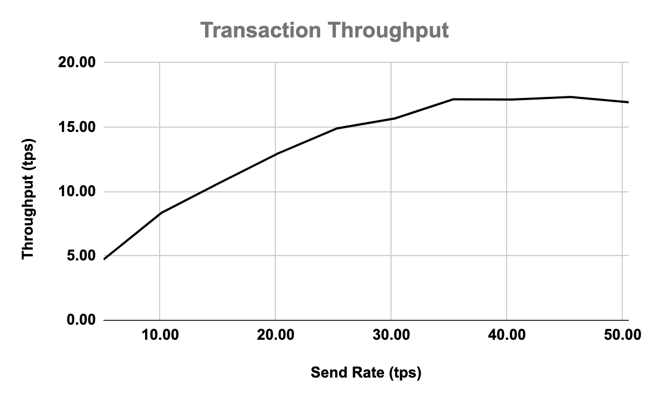 | 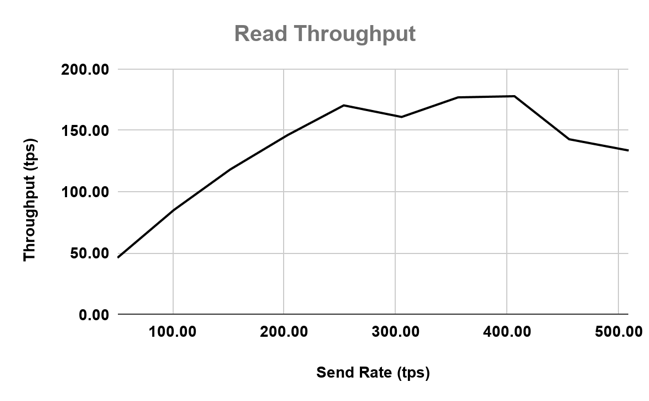 |
| --- | --- |
| Figure 4 Transaction Throughput | Figure 5 Read Throughput |

Figures 6 and 7 illustrate the Transaction Latency and Read Latency, respectively, of the proposed system. The figures present the Maximum Latency (Max), Minimum Latency (Min) and Average Latency (Avg) in seconds (s). We measured the Transaction Latency by changing the send rate from 5 to 50 tps within 10 testing rounds. The average Transaction Latency rose linearly as the sending rate increased (Figure 6). Likewise, we measured the Read Latency by changing the send rate from 50 to 500 tps within 10 testing rounds. The average Read Latency increased slightly as the sending rate was augmented and reached the optimal send rate of 406.50 tps (Figure 7).

| 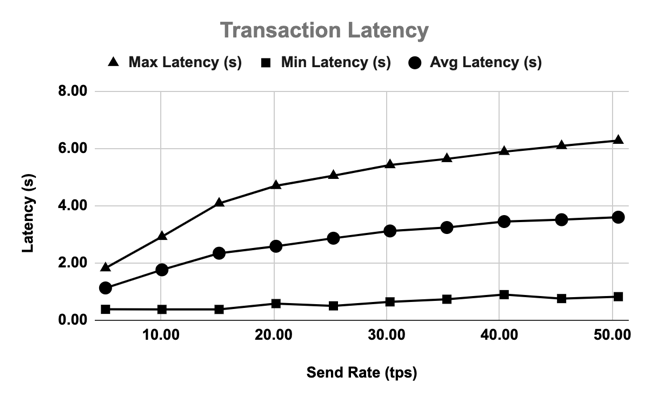 | 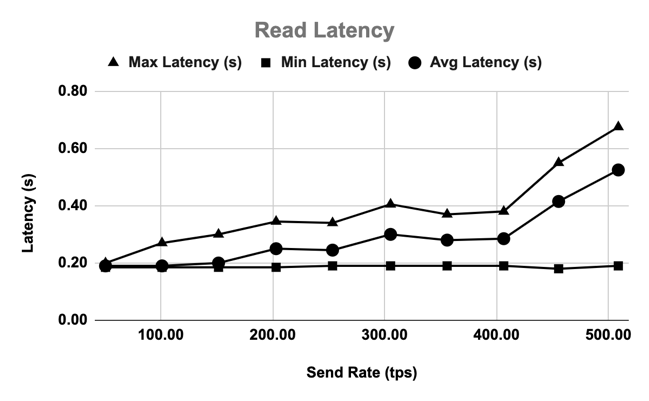 |
| --- | --- |
| Figure 6 Transaction Latency | Figure 7 Read Latency |

Table 12 summarises the results of tests on system scalability. We varied the total number of transactions from 100, 1,000 to 10,000 transactions. These transactions were sent to the blockchain at a linear rate that gradually changes transaction rates from 50 tps to 500 tps during the test to identify transaction rates that affect the system performance. The average of the identified transaction rates was 10.03 tps. As Figure 8 illustrates, the Read throughput was flat in all tests at 10 tps, whereas Transaction throughput was slightly lower at 9 tps, 9.8 tps and 10 tps in tests 1, 2 and 3, respectively. The latency was also flat for Read operations in all tests at 0.19 seconds, while it was higher for Transaction operations at 1.08 seconds, 1.09 seconds and 1.18 seconds. When the number of transactions sent to blockchain increased significantly, Transaction operations latency increased slightly.

| Table 12 Results of system scalability evaluation | | | | |
| --- | --- | --- | --- | --- |
| **Transactions Numbers** | **Transactions Throughput** | **Read Throughput** | **Transactions Latency** | **Read Avg Latency** |
| 100 | 9.1 | 9.9 | 1.11 | 0.19 |
| 1000 | 9.9 | 10 | 1.06 | 0.19 |
| 10000 | 10 | 10 | 1.06 | 0.19= |

**
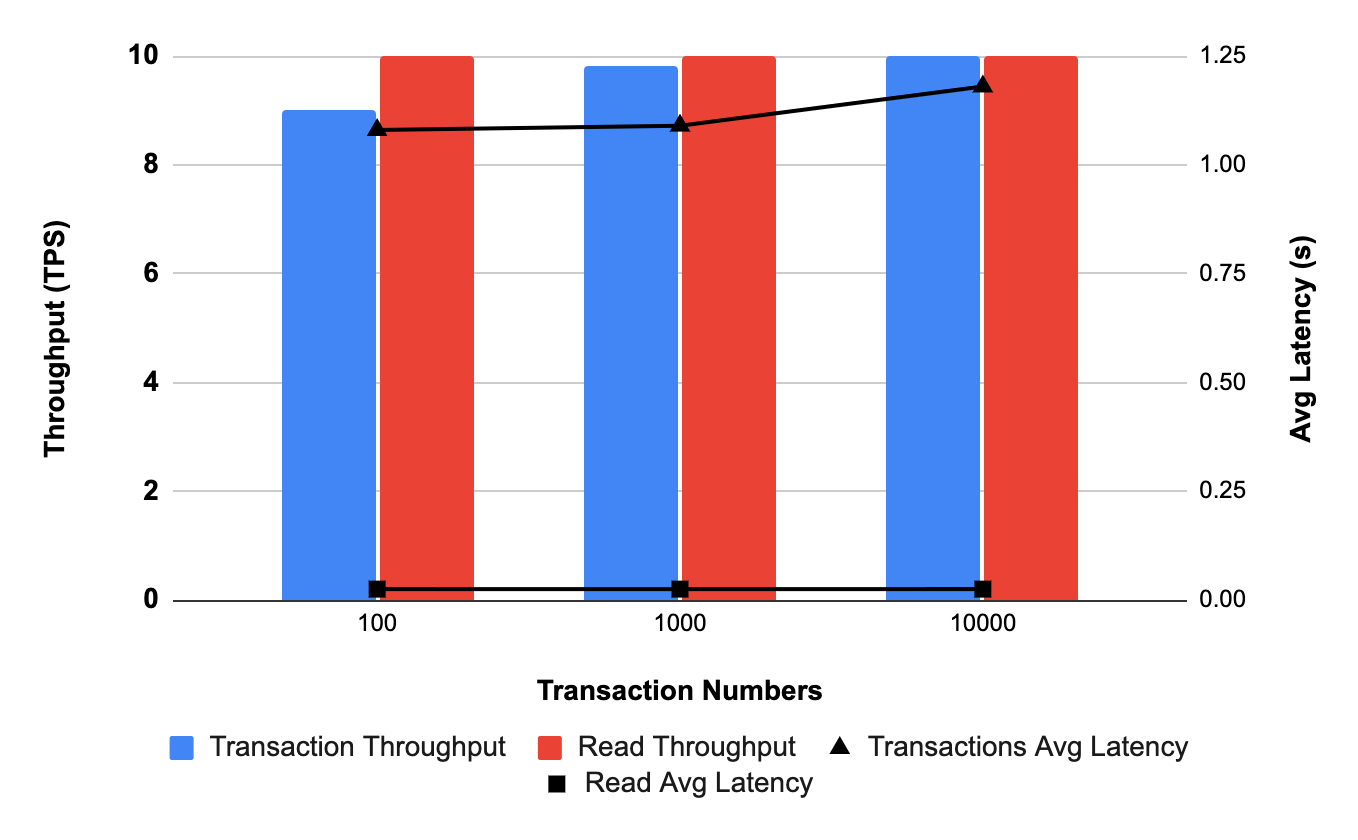
**

Figure 8 The impact of the increasing number of users on throughput and latency for Transaction and Read operations

[1] “Hyperledger Caliper—A Blockchain Benmark Tool.” .

[2] T. H. W. P. W. Group, “Hyperledger Blockchain Performance Metrics,” *Hyperledger.org*. pp. 1–17, 2018, [Online]. Available: https://www.hyperledger.org/wp-content/uploads/2018/10/HL_Whitepaper_Metrics_PDF_V1.01.pdf.

[3] M. Schäffer, M. di Angelo, and G. Salzer, “Performance and Scalability of Private Ethereum Blockchains,” 2019, doi: 10.1007/978-3-030-30429-4_8.
